# Supplementary material for: Quantifying the land and population risk of sewage spills overland using a fine-scale, DEM-based GIS model
Source: PeerJ. 2023 Nov 20;11:e16429. doi: 10.7717/peerj.16429 (PMC10666614; doi:10.7717/peerj.16429)
Supplement: Supplemental Information 1 [file peerj-11-16429-s001.pdf]

APPENDICES

These appendices are split into three sections describing technical components of the paper as well as including supplementary material. The first section, *Data Preprocessing* details the preprocessing steps taken to prepare the datasets for use in the research. These datasets can be found at cited locations and also in part (size permitting) at https://osf.io/hs6g9/. The second section, *LandScan Day Figure*, contains two supplementary maps to Figure 6ac in the *Results* section. The third section, *Development of Figures*, describes the steps to create all the figures throughout the paper.

A. Data Preprocessing

I. Data Collection

To model the sewage spills, it was required to utilize disparate datasets from multiple sources see Table 1. The sewage spills report was scraped from the Georgia Environmental Protection Division (2023). The Georgia Environmental Protection Agency keeps a public record available of the past month's reported sewage spills, including the time, amount discharged, location, and expected cause. The website offers for download the sewage spills for the past 30 days. In order to obtain previous months, it was necessary to scrape the data from the website and then to combine all the scraped records by removing duplicates, but ensuring the most recent version of the spill record is kept. The collection of the other six datasets, LandScan USA 2021: Night (Weber et al., 2021), 3D Elevation Program (3DEP) 1/3 arc-second seamless digital elevation model (DEM) tiles (Weber et al., 2021), TIGER/Line census tracts (U.S. Census Bureau, 2021a), American Community Survey (ACS) 5-year estimates (U.S. Census Bureau, 2021b), National Land Cover Database (NLCD) 2019 Percent Developed Imperviousness (CONUS) (U.S. Geological Survey, 2019), and National Hydrology Dataset (NHD) Plus High Resolution (U.S. Geological Survey, 2022), were retrieved through their respective web portals indicated on the dataset citation. These datasets were used in the risk assessment metrics or in a methodology to prioritize areas for mitigation in a jurisdiction by decision makers.

Table 1: Datasets Information

**Dataset**	**Curator**	**Publication date/range**	**Downloaded**	**Resolution**
Sewage Spills Report	Georgia Environmental Protection Division	2020-05-05 – 2023-01-16	2023-01-17	Closest Address to Spill Location
1/3 Arc-Second Elevation Tiles	U.S. Geological Survey	2022-02-04 – 2022-11-03	2023-01-17	Raster tiles at 1/3 arc-second
Landscan USA 2021 - day & night	Oak Ridge National Laboratory	2022-07-09	2023-01-19	Raster 3 arc-seconds
NHDPlus, subwatershed Boundaries	U.S. Geological Survey	2022-07-06	2023-01-23	1:100,000 scale
TIGER/Line Census Tracts Boundaries	U.S. Census Bureau	2021-10-07	2023-01-20	1:500,000 scale
American Community Survey (ACS) 2021, 5-year Estimates	U.S. Census Bureau	2022-09-15	2023-01-15	N/A
NLCD 2019 Percent Developed Imperviousness (CONUS)	U.S. Geological Survey	2021-06-04	2023-04-26	30 meters

II. Data Cleaning

The sewage spill records contain an overflow address, which were geocoded using the ESRI World Geocoder in ArcGIS Pro 3.0.3. There were significant variations in the way that the addresses were written because these are records collected from a variety of sources. Two differing examples of the overflow address are: "Manhole on the West Side of the Northernmost section of Burnt Hickory Road just south of the railroad at the intersection of Burnt Hickory Road and Hwy 293" and "2414 Stone Road Between Manhole ID C-195 -075 and C-195-073." The geocoder was mostly able to parse the addresses even with these inconsistencies of address input and was able to match 1727 and find ties for 41 of the 1909 records. The average of the geocoding score was 97.03. There were four records that were geocoded outside of the state of Georgia, which were removed from further analysis. Later in the process, in an attempt to mitigate the issues with geocodes, any record that did not meet the following criteria were dropped: the geocode score was under 90, or the geocode's city/state and the spill record city/state did not match. This resulted in 1600 records left. Further, 57 records were dropped because they recorded 0 liters of sewage spilled, meaning that the persons notifying the Georgia Environmental Protection Division were unable to quantify the amount of sewage spilled or the spill was less than 1 gallon.

Of the other six datasets, four had minor data cleaning efforts applied. The 29 3DEP Digital Elevation Model (DEM) tiles that compose the state of Georgia were merged using gdalwarp (see supplementary materials for the specific tiles and the command line arguments at https://osf.io/hs6g9/). For both the elevation raster and the LandScan USA 2021: day raster, we wanted to minimize the amount of records imported into the database, so we utilized the ArcGIS Pro 3.0.3 Extract by Mask tool (ESRI 2023a), where the raster was clipped by the 400-meter buffer surrounding the traceline. The subwatershed layer within the NHDPlus High Resolution dataset was

extracted and all polygons that were within the TIGER/Line Georgia state shape file (U.S. Census Buereau, 2021c) were kept. Some of the subwatershed geometries were "invalid" geometries because of self-intersections, so we utilized the PostGIS function, ST_MakeValid() (PostGIS, 2023c), with default parameters to "make valid" these polygons. Lastly, in the ACS table, we removed excess columns and ensured the formatting of the "Geo_FIPS" column would join with the TIGER/Line census tracts "geoid" column.

III. Data Integration

In order to handle the differences in support of the spatial data, we performed spatial data transformations on two datasets. The LandScan USA 2021: day and the merged elevation raster of the USGS DEMs were transformed into points using ArcGIS Pro 3.0.3 "Raster to Point (Conversion)" tool (ESRI, 2023d). This tool creates a point at the center of the raster cell. Then a buffer is added to the point; for the LandScan points, the buffer size was 35 meters while the DEM points had a buffer of 4.5 meters. These buffer sizes were intentionally chosen to be just slightly smaller than the raster cell size so to count spatial areas only when the polygons intersected a significant portion, so that a spatial unit is not included if only touching a small sliver of the cell area. This choice could mean that buffers around the start and end point are slightly under grouped, or it could also mean that the population counts are less than if joined with the raster. The changing of the support of these raster data also allows for easier spatial joins within PostGIS. The support for the NLCD 2019 Percent Developed Imperviousness raster (U.S. Geological Survey, 2019) also had to be changed to polygons. To do this, Zonal Statistics as Table (ESRI, 2023f) was performed on the raster for each of the subwatershed regions in the Atlanta region. This tool extracted the mean value of imperviousness for the subwatershed region.

B. LandScan Day Figure

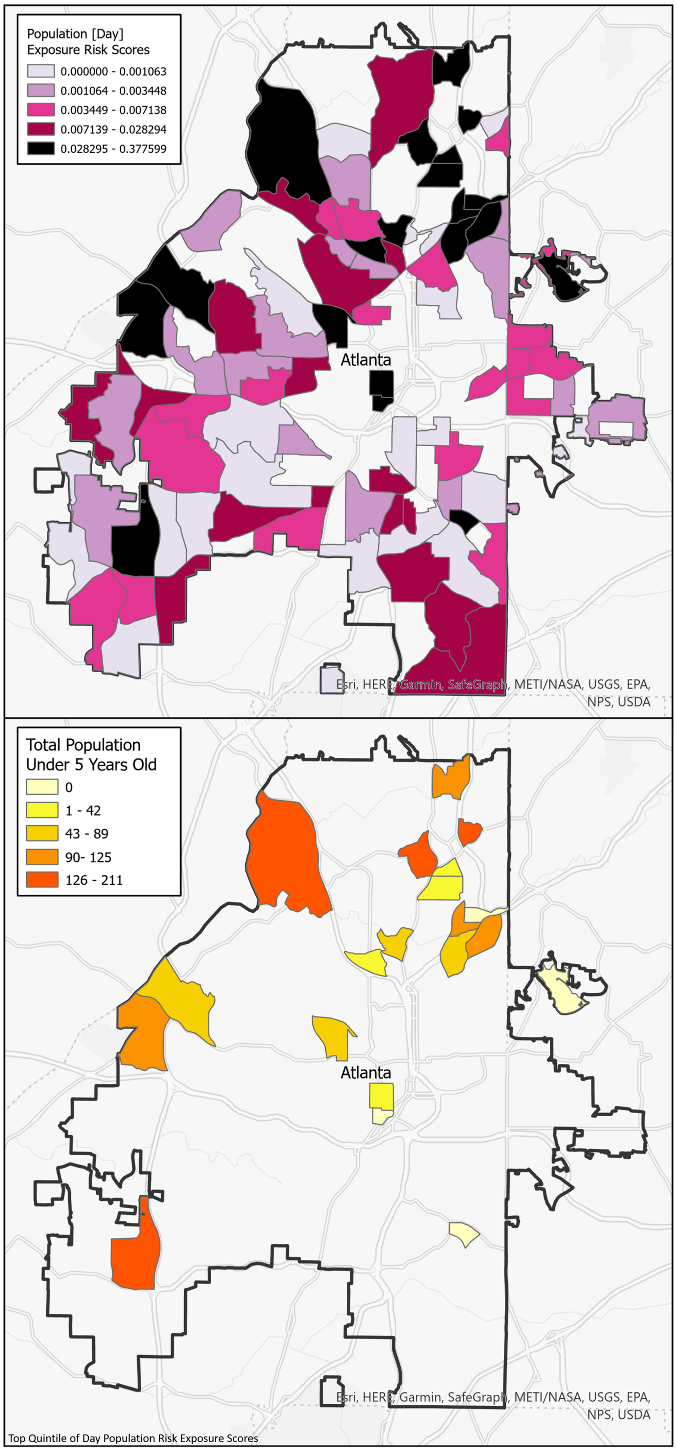

Figure B1: Top figure: Choropleth map of census tracts by population risk metric using night Landscan raster; Bottom figure: Top quintile of population risk map by population under 5 from ACS 2021.

C. Development of Figures

For all maps in figures, ArcGIS Pro 3.0.3 (ESRI (Environmental Systems Research Institute, Inc.), 2022) was utilized. For Figures 2 and 3, we created fake spill locations at high elevation regions of Georgia in order to best illustrate these components of the model. For Figures 4 and 5, we utilized real spill information to create these informative figures. Figure 6a, Public Health Exposure Score for Atlanta was created by clipping all census tracts that were out of the bounds of the Atlanta Place Boundary (U.S. Census Bureau, 2021d). The choropleth map is split by quantiles (evenly split between the 5 classes). The top quintile of Figure 6a, was utilized to create Figure 6b. These census tracts were joined with the ACS 2021 columns, Total: Male: Under 5 Years "ACS21_5yr_B01001003" and Total: Female: Under 5 Years "ACS21_5yr_B01001027." Figure 6c was created by clipping all the subwatershed regions boundaries by the Atlanta Place Boundary (U.S. Census Bureau, 2021d). The choropleth map is split by quantiles, or evenly split between the 5 classes. To create the imperviousness percentage for subwatersheds map, Figure 6d, the raster underneath the polygons was summarized by subwatershed region using ArcGIS Pro's Zonal Statistics tool, which gets the mean of the raster cells for each of the subwatershed regions in the Atlanta region. The classes (seen in the legend) are split by the quantile method (evenly split). Figure B1ab was created just like Figure 6ac, but with the Day Landscan. Figure 7a was created by intersecting the top quintile from Figure 6a and 6c. The pins are the centroids of the intersection geometries. Figure 7b was created by overlaying the text of the summed quantity of the 5 spills that occurred at this location (662,123). Next to this model we took screen shots from Google Streetview (Google Maps, 2022) of the small urban farm across the street. We anonymized the location so not to impact the community farm.
